# Electroencephalography microstate alterations reflect potential double‐edged cognitive adaptation in Ménière's disease

**DOI:** 10.1111/cns.14896

**Published:** 2024-08-06

**Authors:** Yi‐Ni Li, Jie Li, Peng‐Jun Wang, Dong‐Zhen Yu, Zheng‐Nong Chen, Zheng‐Yu Shi, Ya‐Qin Wu, Wei‐Dong Qi, Wen Lu, Hai‐Bo Shi

**Affiliations:** ^1^ Department of Otolaryngology‐Head and Neck Surgery Shanghai Sixth People's Hospital Affiliated to Shanghai Jiao Tong University School of Medicine Shanghai China; ^2^ Department of Otolaryngology Head and Neck Surgery Huashan Hospital Fudan University Shanghai China

**Keywords:** cognitive adaptation, EEG, Ménière's disease, microstate, postural control

## Abstract

**Purpose:**

To explore the microstate characteristics and underlying brain network activity of Ménière's disease (MD) patients based on high‐density electroencephalography (EEG), elucidate the association between microstate dynamics and clinical manifestation, and explore the potential of EEG microstate features as future neurobiomarkers for MD.

**Methods:**

Thirty‐two patients diagnosed with MD and 29 healthy controls (HC) matched for demographic characteristics were included in the study. Dysfunction and subjective symptom severity were assessed by neuropsychological questionnaires, pure tone audiometry, and vestibular function tests. Resting‐state EEG recordings were obtained using a 256‐channel EEG system, and the electric field topographies were clustered into four dominant microstate classes (A, B, C, and D). The dynamic parameters of each microstate were analyzed and utilized as input for a support vector machine (SVM) classifier to identify significant microstate signatures associated with MD. The clinical significance was further explored through Spearman correlation analysis.

**Results:**

MD patients exhibited an increased presence of microstate class C and a decreased frequency of transitions between microstate class A and B, as well as between class A and D. The transitions from microstate class A to C were also elevated. Further analysis revealed a positive correlation between equilibrium scores and the transitions from microstate class A to C under somatosensory challenging conditions. Conversely, transitions between class A and B were negatively correlated with vertigo symptoms. No significant correlations were detected between these characteristics and auditory test results or emotional scores. Utilizing the microstate features identified via sequential backward selection, the linear SVM classifier achieved a sensitivity of 86.21% and a specificity of 90.61% in distinguishing MD patients from HC.

**Conclusions:**

We identified several EEG microstate characteristics in MD patients that facilitate postural control yet exacerbate subjective symptoms, and effectively discriminate MD from HC. The microstate features may offer a new approach for optimizing cognitive compensation strategies and exploring potential neurobiological markers in MD.

## Graphical abstract

We identified several EEG microstate characteristics in MD patients, which facilitate postural control yet exacerbate subjective symptoms and effectively discriminate MD from HC. Microstate features may serve as a new avenue for optimizing cognitive compensation strategies and exploring potential neurobiological markers in MD.

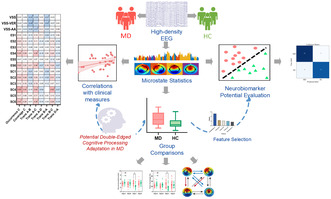

## 1 INTRODUCTION

Meniere's Disease (MD) is a multifactorial inner ear disorder with unpredictable episodes and variable severity, characterized by recurrent spontaneous vertigo, fluctuating sensorineural hearing loss, tinnitus, and aural fullness.^1^, ^2^ With a prevalence ranging from 3 to 513 per 100,000 individuals, MD may present in a variety of ways and experience diverse natural progression, posing challenges for diagnosis and treatment evaluation.^2^, ^3^, ^4^

As a lead to the loss of quality‐associated life‐years, patients with MD commonly experience gait and balance impairment and, in severe cases, may suffer from “drop attack,” where sudden distortion of vertical orientation leads to a fall without loss of consciousness.^2^ Various vestibular testing has been considered to have a supportive role,^1^, ^5^, ^6^ but the heterogeneity of the results and the partial inconsistency with subjective symptoms suggest that new assessment methods and further insights into the pathophysiology of balance impairment from additional perspectives may be needed.

Considering the crucial role of multi‐sensory convergence and sensorimotor integration in adaptable postural and balance control,^7^ central alterations has been raised as a potential direction for further understanding the balance complaints especially in chronic otogenic vertigo patients.^8^, ^9^, ^10^, ^11^, ^12^ Previous studies reported a decreased volume of hippocampus in patients with MD, which correlated with the severity of hearing and vestibular loss.^13^, ^14^ In addition to the structural investigations indicating an impact of MD in brain areas, the development of functional neuroimaging tools has provided more opportunities to understand the dynamic neural function in MD. Despite the wide application of various neuroimaging approaches in neurological and psychological areas, there were relatively few studies focused on the intrinsic functional networks and neural activity in MD patients. Recently, a resting‐state functional magnetic resonance imaging (rs‐fMRI) study reported the functional reorganization of intranetwork and internetwork connectivity in MD patients, manifesting as a decreased functional connectivity in the somatomotor, auditory, ventral attention, default mode, limbic, and deep gray matter networks.^10^ The correlations between some altered brain network indexes and the clinical stages suggested that large‐scale network reorganizations revealed by neuroimaging techniques could provide further understanding of MD's pathology. However, the indicators correlated with the severity of clinical complaints and balance performance have yet to be investigated.

Comparing to other neuroimaging tools, the high temporal resolution, comparatively lower cost, adaptability to different conditions, and convenience in acquisition of electroencephalography (EEG) provide it more chance for clinical applications. Microstates are non‐overlapping and quasi‐stable topography patterns of EEG scalp potential fields which reveals dynamical activity of large‐scale brain networks on a subsecond timescale.^15^, ^16^ Comparing to rs‐fMRI which focuses on the strength of relationships among cortical networks, EEG microstates provide more information about their patterns of temporal activity.^16^, ^17^ Recently, the EEG microstate analysis has achieved fascinating results in numerous neurological and psychological diseases,^18^, ^19^, ^20^, ^21^, ^22^ showing huge potential as biomarkers or phenotype candidates for various neurological conditions.

Currently, to the best of our knowledge, the microstate profiles of patients with MD remain unknown. The aim of our study was to identify the altered microstate pattern of patients with MD, explore its clinical significance, and test whether these microstate characteristics could potentially be the biomarker for MD. In order to get more reproducible and explainable results, the four canonical classes of microstates were taken in the present study. A detailed correlation analysis with balance performance and vertigo severity was conducted to further interpret the underlying neural mechanism of postural control. Finally, a machine learning classifier was established to evaluate how well the microstate profiles may serve as a biomarker for MD by distinguishing patients from healthy participants. The results showed that the temporal dynamics of microstates class C and the syntax of class A could be candidate neural signatures for MD. The findings shall provide insights into the neurobiological mechanisms of balance impairment in MD and a potential feature to facilitate clinical diagnosis.

## 2 METHODS

### 2.1 Participants

A total of 32 patients diagnosed with MD were recruited from the Otolaryngology‐Head and Neck Surgery Department of the Shanghai Sixth People's Hospital Affiliated to Shanghai Jiao Tong University School of Medicine. The clinical diagnosis of MD is established by experienced professional doctors according to the criteria outlined by the Bárány Society. We also recruit 29 age‐ and gender‐matched healthy controls (HC) with no history of vertigo attacks.

**FIGURE 1:**
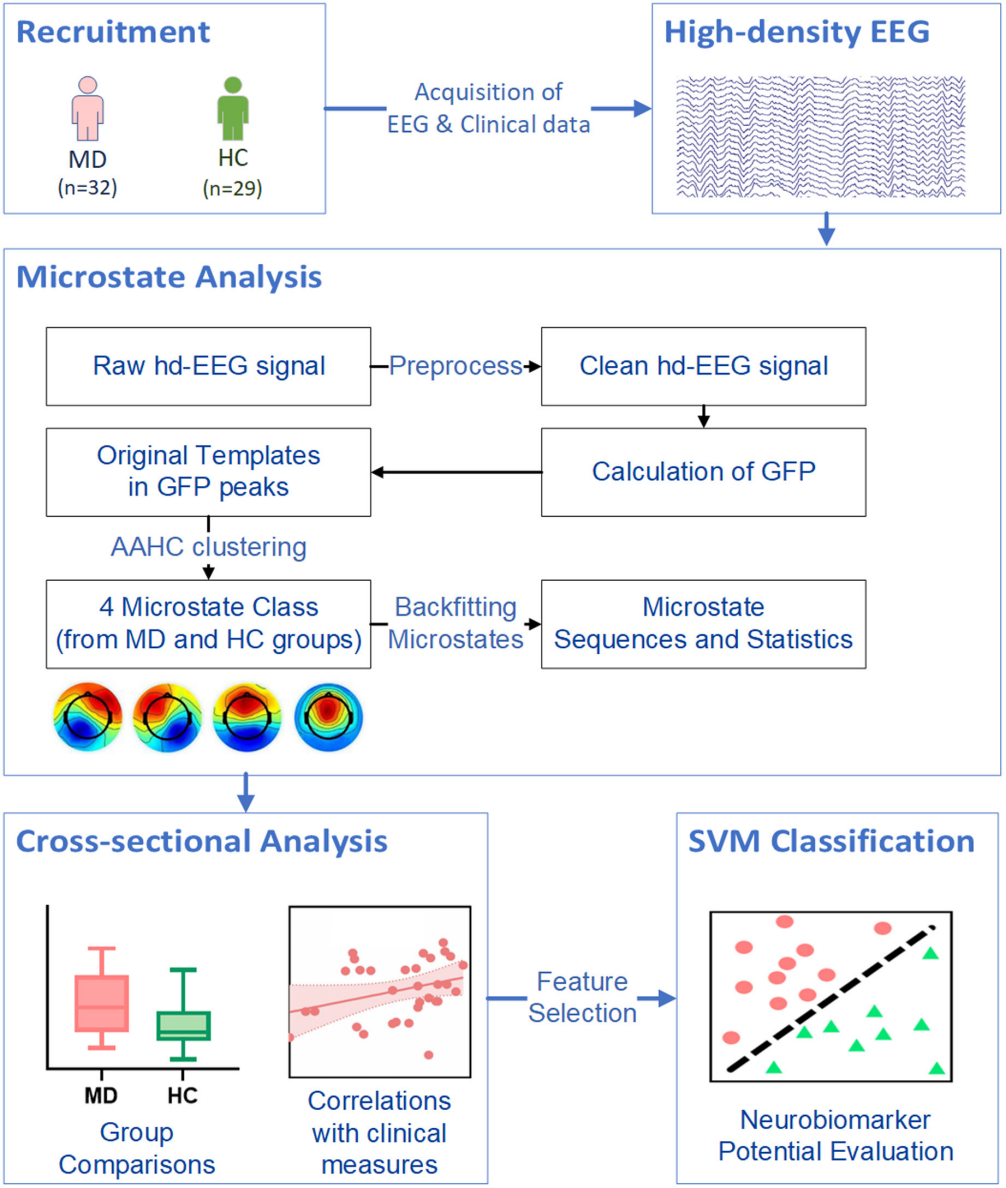
Overview of the main steps of analysis. Following the preprocessing of hd‐EEG data, we calculated the GFP for each participant. The topologies at local maxima of the GFP were subjected to the AAHC algorithm, resulting in four microstate templates. After backfitting the group maps to the original data, we computed the microstate statistics based on the microstate sequences. We then performed a cross‐sectional comparison of the microstate characteristics between MD patients and HC participants and further analyzed the correlations between microstate characteristics and clinical measures in MD patients. The significant characteristics identified through cross‐section analysis were subsequently fed into the SVM classifier to evaluate the potential of microstate characteristics as future neurobiomarkers.

The exclusion criteria for both groups included: (1) age below 18 years old; (2) inability to complete the questionnaire and vestibular function tests with the help of doctors and researchers, including MD patients during acute attacks; (4) a current or long‐term history of substance dependence; (5) comorbid with severe systemic diseases, psychiatric disorders or neurological disorders (i.e., encephalitis, epilepsy). All participants were right‐handed and provided written informed consent after a detailed explanation. This study was approved by the Institutional Ethics Review Board of the Shanghai Sixth People's Hospital Affiliated to Shanghai Jiao Tong University School of Medicine (Approval No: 2020‐128). A schematic overview of the research is shown in Figure 1.

### 2.2 Clinical assessment

We used the vertigo symptom scale (VSS) to evaluate the severity of vestibular symptoms, including the frequency of dizziness, vertigo, imbalance, and related autonomic symptoms during the past 12 months. It contains two subscales, the anxiety and autonomic symptom scale (VSS‐AA) and the vertigo symptom scale (VSS‐VER).^23^ In addition, the 9‐item patient health questionnaire subscale (PHQ‐9)^24^, ^25^ and the 7‐item generalized anxiety disorder scale (GAD‐7)^26^ were employed to assess depression and anxiety, respectively.^27^

Pure‐tone audiometry was obtained at 0.25–8 kHz (GSI‐61; Grason‐Stadler, Inc.) and the pure‐tone average (PTA) was calculated as the simple arithmetic means of 0.5, 1, and 2 kHz pure‐tone threshold. Staging was determined based on the worst recorded PTA during intermittent periods over the past 6 months: Stage I is defined as a PTA of ≤25 dBHL, Stage II as 26–40 dBHL, Stage III as 41–70 dBHL, and Stage IV as >70 dBHL.^28^

The computerized dynamic posturography (CDP) was used to evaluate the balance performance and postural stability (Equitest, NeuroCom International, Inc., Clackamas, OR, United States). The equilibrium composite score (EC) of the whole sensory organization test (SOT) was derived from six individual equilibrium scores (ES), obtained in six progressively challenging conditions subjectively.^29^ Eyes were instructed to be closed in condition 2 and 5, while the support platform was sway‐referenced in condition 3 to 6. The visual surround was also sway‐referenced in condition 3 and 6. The ability to use somatosensory (SOM), visual (VIS), and vestibular system (VEST) to maintain postural control was measured by ratios, respectively ES2/ES1, ES4/ES1, and ES5/ES1. The ability to inhibit misuse of false visual information (PREF) was calculated by (ES3 + ES6)/(ES2 + ES5).^27^, ^30^ In each condition, the strategy score (SC) quantified the amount of ankle and hip movement, with zero indicating a hip‐dominant strategy and 100 indicating an ankle‐dominant strategy.

### 2.3 Electroencephalography data acquisition and preprocessing

A 256‐channel system (Electrical Geodesics, Eugene, OR, United States) was used to acquire high‐density EEGs (hd‐EEG) with a sampling rate of 1000 Hz. Participants were instructed to sit comfortably in a sound‐shield room, to remain relaxed and awake, and to minimize eye‐movement throughout the recording. Resting‐state EEGs were recorded for 7 min in both eyes‐closed and eyes‐open paradigm. Only eyes‐closed EEG data were analyzed. Custom MATLAB (Mathworks, Massachusetts, USA) scripts based on EEGlab toolbox^31^ (Delorme and Makeig, 2004) were used for offline preprocessing. The data were filtered with a bandpass of 0.5–200 Hz, downsampled to 500 Hz, topographically interpolated bad channels, segmented into 2‐s segments, and re‐referenced to the grand average. The eye movement, heartbeat, and muscle artifacts were removed by independent component analysis (ICA).

### 2.4 Microstate analysis

Consistent with previous work, the microstate analysis was conducted using the Microstate Analysis plugin (Version 0.3, Thomas König, University of Bern, Switzerland) for EEGLAB. After a band‐pass filter between 2 and 20 Hz, the global field power (GFP) was calculated. The local maxima of the GFP curve reflect the moments of highest field intensity and highest topographical signal to noise ratio. Thus we submitted only EEG topographies at the GFP peaks to the “atomize and agglomerate hierarchical clustering” (AAHC) algorithm and figure out the four most dominant template maps ignoring polarity inversion.^15^, ^32^ The group templates were further calculated separately through a second AAHC cluster analysis across the individual template maps of each subject. Base on the spatial correlation, we back fit the group template into each individual data and calculated the global explained variance to evaluate how well our templates explained the EEG topography throughout the time series.

At last, we calculated the classical dynamic characteristics for each microstate class: mean duration, time coverage, frequency of occurrence, and transitions between each couple of microstate classes. Mean duration (ms) is the average duration of time in which a given microstate remains constant. Time coverage (%) is the percentage of the total analysis time spent in a given microstate, also known as “Contribution.” Occurrence is the average number of times per second that a specific microstate becomes dominant.^15^, ^16^, ^33^

### 2.5 Machine learning

To investigate the potential of microstate features to differentiate MD patients from HC, we established a support vector machine (SVM) classifier with a linear kernel based on the microstate and demographic features. The feature selection was followed a backward strategy. Considering our limited sample size, we obtained the machine parameters and performance estimation via a nested leave‐one‐out cross‐validation framework. The penalty parameter C was optimized by a fivefold grid search process. We computed the accuracy, sensitivity, specificity, and balanced accuracy to measure its performance. The balanced accuracy was calculated as the average of sensitivity and specificity. To avoid potential problems arising from the lack of independence, we further conducted a permutation test with 5000 iterations to verify the significance of our model. Machine learning was performed with Scikit‐learn in Python (version 3.8.8).

### 2.6 Statistical analysis

Statistical tests were performed with IBM SPSS 26 (IBM Corp., Chicago, USA) and GraphPad Prism Software (San Diego, CA). Shapiro–Wilk's normality tests were performed for continuous variables. Continuous variables were reported as mean ± standard deviation or median (interquartile range) according to their distribution. Unpaired *t*‐test and Mann–Whitney test were used to compare continuous variables, depending on their distribution. Nonparametric chi‐squared test was employed to compare gender ratio. To assess between‐group differences in microstate parameters, an analysis of two‐way analysis of variance (ANOVA) with post hoc group comparisons was applied. The Group and Microstate Class were taken as factors. Bonferroni's correction was used for correcting multiple comparisons. A p level of less than 0.05 (two‐sided) was considered to be statistically significant. Correlation between two variables was determined by Spearman correlation.

## 3 RESULTS

### 3.1 Demographics and basic characteristics

As presented in Table 1, there was no statistically significant difference between MD patients and HC in terms of demographic characteristics such as age (*t* = 0.5723, *p* = 0.5693), gender (*x* ^2^ = 0.1250, *p* = 0.7237), and education year (*U* = 440, *p* = 0.7239). As expected, MD patients demonstrated a significant increase in PTA thresholds (*t* = 3.247, *p* = 0.0019). However, despite significant complaints of vertigo symptoms (*U* = 41, *p*<0.0001), no significant differences were observed in the ECs of computerized dynamic posturography (*t* = 0.5750, *p* = 0.5675). Consistent with previous studies, MD patients also obtained significantly higher scores on PHQ9, GAD7, and PSQI scales.

**TABLE 1:** General demographic and clinical characteristics.

	MD (*n* = 32)	HC (*n* = 29)	Group comparison
Age, years, mean ± SD	54.75 ± 11.93	56.69 ± 14.52	*t* = 0.5723, *p* = 0.5693
Gender (F/M)	23/9	22/7	*x* ^2^ = 0.1250, *p* = 0.7237
Education, years, median (IQR)	12 (6)	12 (3)	*U* = 440, *p* = 0.7239
VSS, median (IQR)	23.5 (25)	2 (6.5)	*U* = 41, *p*<0.0001****
PHQ‐9, median (IQR)	6 (8.5)	2 (6.5)	*U* = 260.5, *p* = 0.0026**
GAD‐7, median (IQR)	4.5 (7)	0 (2)	*U* = 207.5, *p*<0.0001****
ECs, mean ± SD	69.66 ± 8.36	70.90 ± 8.47	*t* = 0.5750, *p* = 0.5675
PTA, mean ± SD	45.94 ± 25.29	27.14 ± 17.58	*t* = 3.247, *p* = 0.0019**
History of vertigo (years)	3.96 ± 6.69		

*Note*: *p < 0.05, **p < 0.01, ***p < 0.001, ****p < 0.0001.

### 3.2 Microstate topographies and dynamic parameters

**FIGURE 2:**
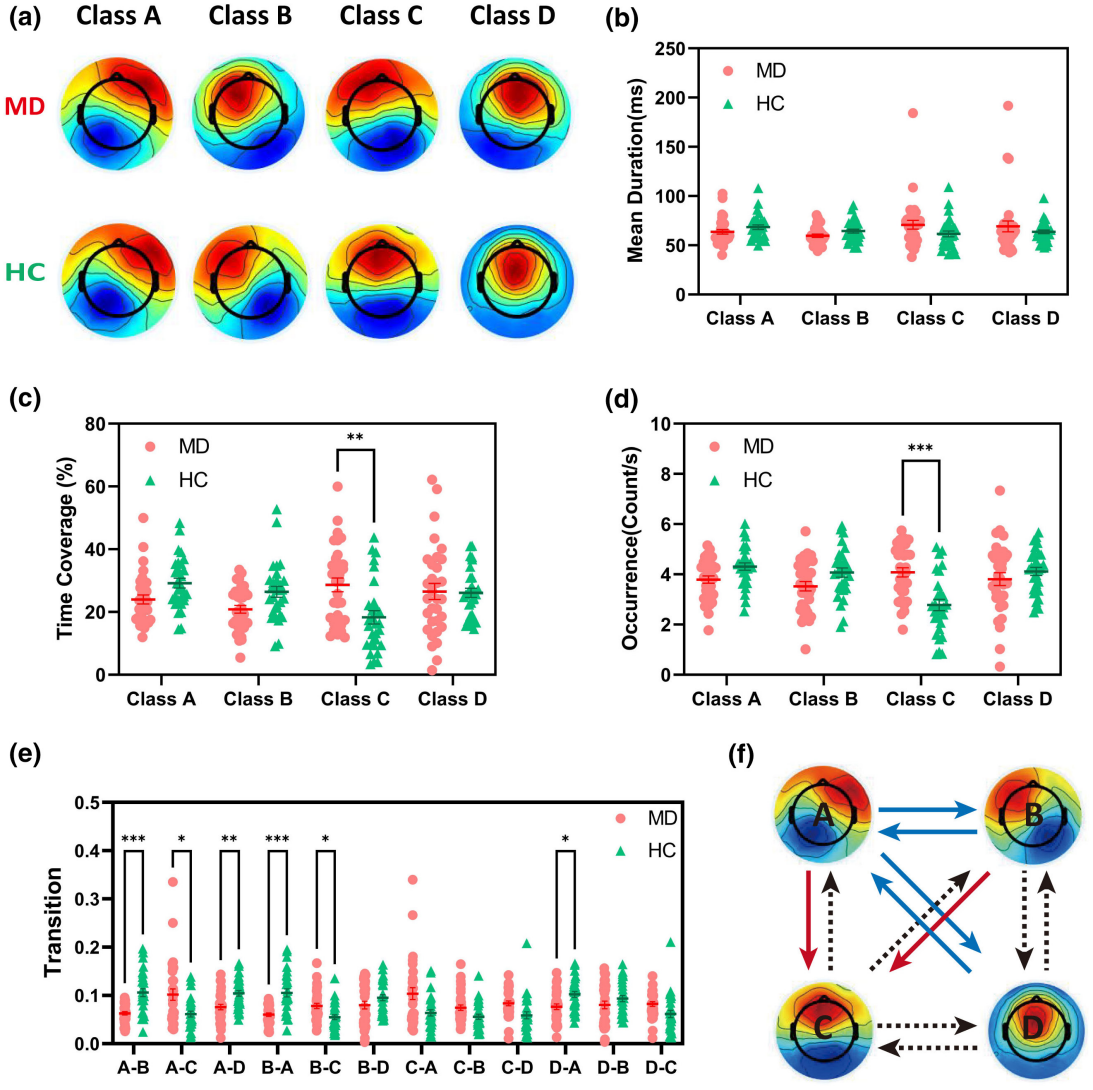
The spatial configuration and dynamic characteristics of microstate classes. (a) Global architecture of the four microstate classes, separately for MD patients and HC participants. (b–d) Group average temporal parameters, (b) mean duration, (c) time coverage, and (d) occurrence frequency, of each class for MD patients versus HC participants. (e, f) Microstate syntax analysis results. (e) Group average transitions and group comparisons. (f) Schematic view of microstate syntax. Data of MD patients are displayed in red dots and HC in green triangles. Significant increases in MD are represented by solid red arrows, significant decreases by solid blue arrows, and no difference by dashed arrows. Error bars represent SEM. *p < 0.05, **p < 0.01, ***p < 0.001 by post hoc Bonferroni's multiple comparisons tests.

As shown in Figure 2a, we identified four dominant microstate classes labeled as A, B, C, and D, resembling classic templates commonly reported in prior studies. These classes, respectively, explained 78.38% and 77.07% of the global variance in MD patients and HC participants.

The Group average statistics of mean duration, time coverage, and frequency of occurrence are depicted in Figure 2b–d. Two‐way repeated measures (rm) ANOVAs showed significant Microstate Class × Group interaction for time coverage (*F* = 6.023, *p* = 0.0006) and occurrence (*F* = 10.70, *p* < 0.0001), indicating a dependence of group difference on microstate class. No significant interaction was detected for mean duration (*F* = 2.432, *p* = 0.0667). Post hoc tests (Table 2) showed that MD patients had increased occurrence (*p* = 0.0001) and time coverage (*p* = 0.0049) of class C.

**TABLE 2:** Comparison of microstate dynamic parameters.

Microstate	MD	HC	Adjusted *p* value
Mean duration (ms)			
Class A	63.56 ± 13.08	68.39 ± 12.69	*p* = 0.5977
Class B	59.71 ± 9.29	64.39 ± 10.64	*p* = 0.2951
Class C	70.77 ± 25.48	61.64 ± 15.76	*p* = 0.3798
Class D	69.17 ± 30.98	63.70 ± 10.36	*p* > 0.9999
Occurrence (/s)			
Class A	3.79 ± 0.81	4.31 ± 0.78	*p* = 0.0552
Class B	3.52 ± 1.07	4.07 ± 0.99	*p* = 0.1693
Class C	4.08 ± 1.04	2.78 ± 1.20	*p* = 0.0001***
Class D	3.81 ± 1.44	4.12 ± 0.89	*p* > 0.9999
Time coverage (%)			
Class A	23.97 ± 8.10	29.19 ± 8.32	*p* = 0.0647
Class B	20.86 ± 7.06	26.43 ± 9.46	*p* = 0.0501
Class C	28.64 ± 12.39	18.28 ± 11.43	*p* = 0.0049**
Class D	26.52 ± 14.57	26.09 ± 7.78	*p* > 0.9999

*Note*: *p < 0.05, **p < 0.01, ***p < 0.001, ****p < 0.0001.

As for the microstate syntax, the transitions are depicted in Table 3. Compared to the HC group, MD patients showed fewer transitions between class A and B (both, *p* < 0.001) and between class A and D (both, *p* < 0.05). In addition, transitions from class A to C (*p* = 0.0459) and from class B to C (*p* = 0.0365) both increased in MD patients (Figure 2e,f).

**TABLE 3:** Comparison of transition probabilities.

Transition	MD	HC	Adjusted *p* value
A to B	0.06 ± 0.02	0.11 ± 0.04	*p* = 0.0003***
A to C	0.10 ± 0.07	0.06 ± 0.03	*p* = 0.0459*
A to D	0.08 ± 0.03	0.10 ± 0.03	*p* = 0.0094**
B to A	0.06 ± 0.02	0.11 ± 0.04	*p* = 0.0001***
B to C	0.08 ± 0.03	0.05 ± 0.03	*p* = 0.0365*
B to D	0.08 ± 0.05	0.10 ± 0.03	*p* > 0.9999
C to A	0.10 ± 0.07	0.06 ± 0.03	*p* = 0.0717
C to B	0.07 ± 0.03	0.06 ± 0.03	*p* = 0.2142
C to D	0.08 ± 0.03	0.06 ± 0.04	*p* = 0.0787
D to A	0.08 ± 0.03	0.10 ± 0.03	*p* = 0.0392*
D to B	0.08 ± 0.05	0.09 ± 0.03	*p* > 0.9999
D to C	0.08 ± 0.03	0.06 ± 0.04	*p* = 0.3135

*Note*: *p < 0.05, **p < 0.01, ***p < 0.001, ****p < 0.0001.

### 3.3 Correlation between microstate and clinical characteristics

**FIGURE 3:**
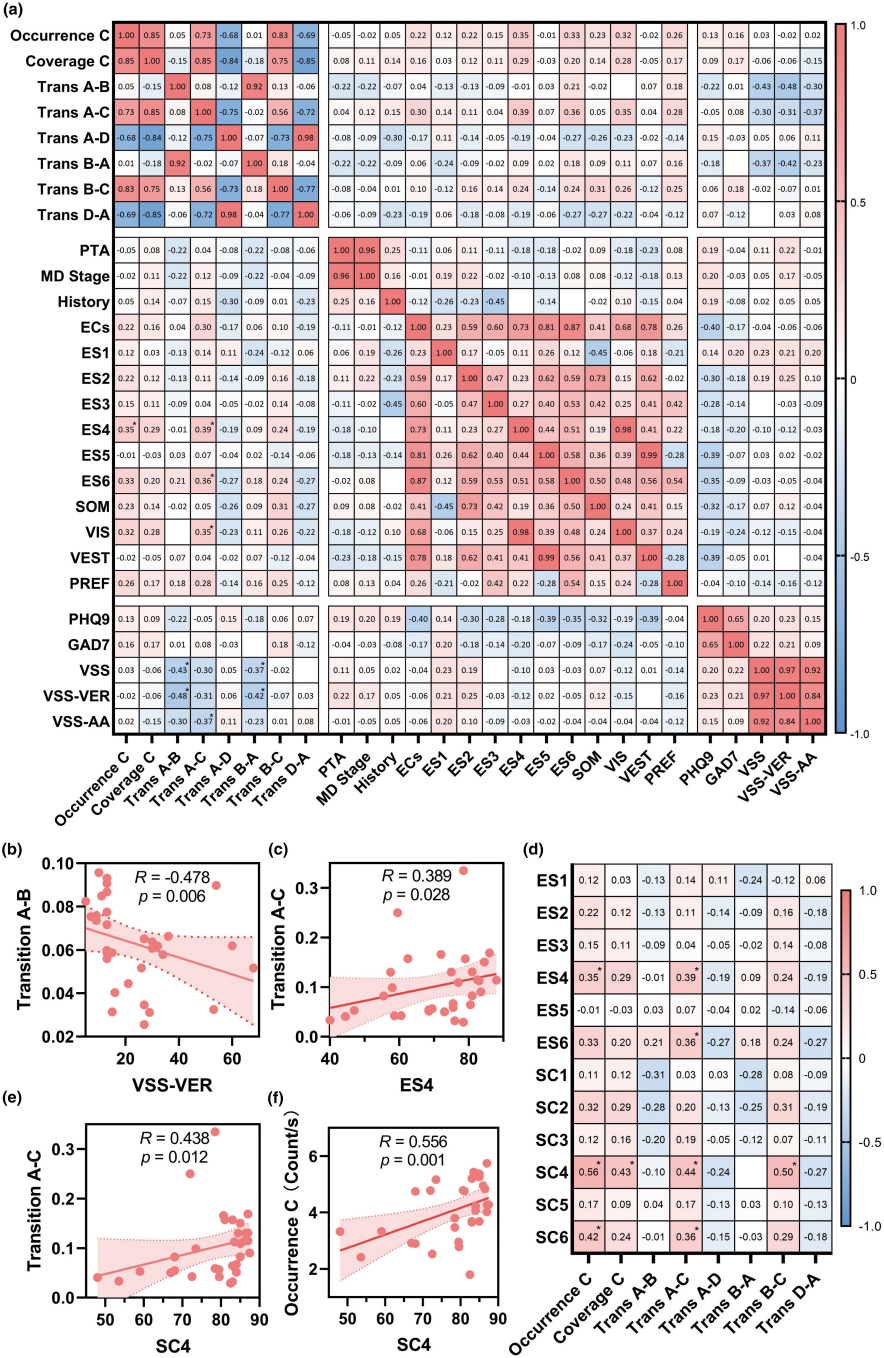
Correlations between microstates and clinical characteristics in MD patients. (a) Correlation matrix between differential microstate and clinical characteristics. (b) Scatterplot of transitions from class A to B and vestibular subscales of VSS. (c) Scatterplot of transition from class A to C and ES in condition 4. (d) Correlation matrix between differential microstate characteristics and both equilibrium and strategy scores. (e, f) Scatterplot of SC in condition 4 and transitions from class A to B (e), occurrence frequency of class C (f). Spearman *R* values are marked. *p < 0.05. PTA, pure‐tone average; ECs, equilibrium composite scores; ES1‐6, equilibrium score in condition 1–6; SOM, somatosensory ratios; VIS, visual ratios; VEST, vestibular ratios; PREF, visual preference; VSS, vertigo symptom scale; VSS‐VER, vertigo symptom scale in VSS; VSS‐AA, autonomic symptom scale in VSS; SC1‐6, sensory strategy scores in condition 1–6.

Our correlation analysis revealed no statistically significant association between microstate dynamics and PTA, duration of history, PHQ‐9 nor GAD7. However, VSS scores were negatively correlated with the transition between class A and B (*p* = 0.0138, *R* = −0.431; *p* = 0.0379, *R* = −0.369) (Figure 3a). To prevent false positives caused by the impact of autonomic symptoms, we further confirmed the correlations between microstate characteristics and vestibular subscale of VSS (*p* = 0.0057, *R* = −0.478; *p* = 0.0164, *R* = −0.421) (Figure 3a,b). Additionally, the transition from class A to C was positively correlated with the equilibrium score in condition 4 (*p* = 0.0277, *R* = 0.389) and condition 6 (*p* = 0.0405, *R* = 0.364). Thus it was also positively correlated with the overall score for visual system (*p* = 0.0493, *R* = 0.350).

Considering the associations between microstate dynamics and balance performance in challenging conditions revealed by CDP, we further performed another correlation analysis to explore the relationship between microstate dynamics and strategy scores. The occurrence of class C as well as transitions from A to C turned out to be positively correlated with SC in both condition 4 and condition 6 (Figure 3d,e), while the time coverage of class C and transitions from B to C were only positively correlated with SC in condition 4 (Figure 3d,f).

### 3.4 Classification Performance in SVM classifier based on microstate characteristics

**FIGURE 4:**
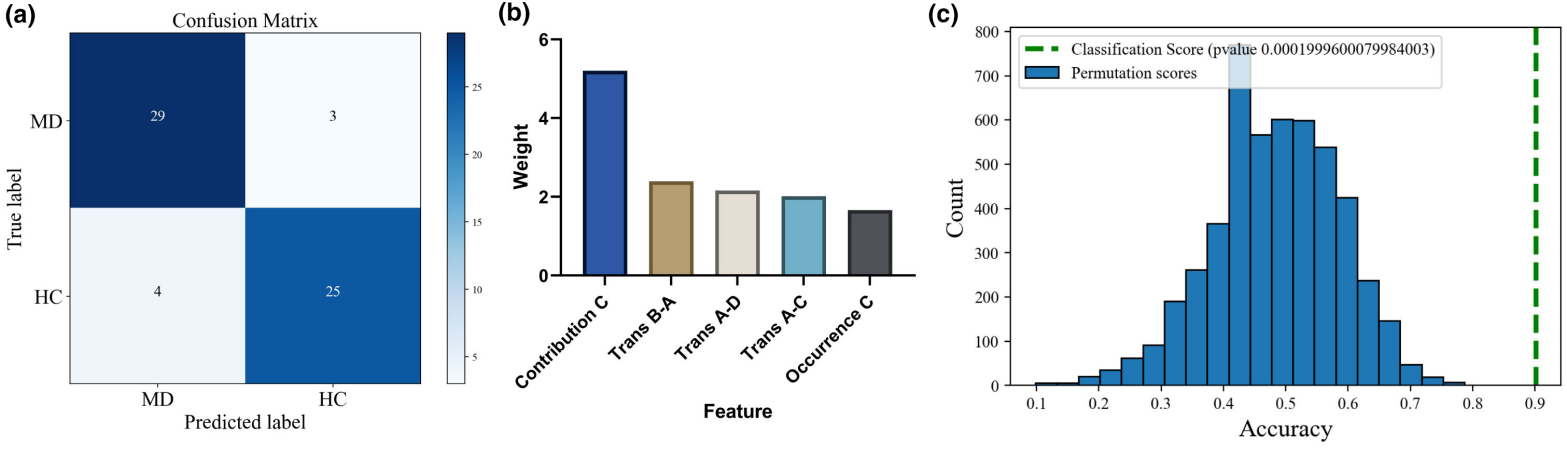
Performance of SVM classifier based on microstate and demographic parameters. (a) Confusion matrix of SVM classification. (b) Feature weights of the five most important features in the classifier. (c) Post hoc test for SVM model via permutation testing with 5000 iterations.

With feature reduction and model optimized by a backward strategy, the linear SVM classifier based on demographic characteristics and seven microstate features could differentiate MD patients from HC participants with an accuracy of 88.52%, a sensitivity of 86.21%, and a specificity of 90.61%. The confusion matrix and the weights of the five most important features are presented in Figure 4. The statistical significance of this model was further confirmed via permutation testing (*p* = 0.0002).

## 4 DISCUSSION

The present study investigated the brain dynamics in MD patients with an EEG microstate analysis, which demonstrated alterations in the pattern of neural network transitions on a sub‐second timescale. The distinct features in MD patients, primarily characterized by increased frequency of class C and syntax alterations of class A, showed significant correlations with both balance performance and vertigo symptoms. It also provided discriminative features to distinguish MD patients from HC participants, indicating a potential role of microstate signatures for clinical diagnosis and assessment.

In our study, microstate class C exhibited higher time coverage and occurrence in MD patients compared to HC participants. According to previous studies, microstate class C is associated with the saliency network (SN), reflecting activation in the dorsal anterior cingulate cortex, inferior frontal cortices, and anterior insula.^15^, ^34^, ^35^ Our results regarding class C indicate more frequent but stable activations of the SN in MD patients during the resting state. As we know, the SN plays an important role in identifying the most homeostatically relevant stimuli among multiple competing external and internal stimuli,^36^ acting as a gatekeeper of executive control and showing increased activation in stimulus‐driven cognitive processing.^37^ Executive function, especially inhibitory control, has been reported to play a crucial role in dynamic balance control and postural adjustment.^38^ With fluctuating vestibular dysfunction in MD patients, the increases in microstate class C probably represent a compensation strategy to handle the competing vestibular information with greater involvement of cognitive processing.

In the correlation analysis between microstate characteristics and ESs in challenging conditions from condition 3 to 6, the increase in occurrence frequency of class C showed a positive correlation with ES in condition 4 (Figure 3a,d). The further correlation analysis with strategy scores showed a similar positive correlation in both condition 4 and condition 6 (Figure 3d, f). In condition 4, the platform was sway‐referenced with visual surround stable and the eyes open, providing conflicting somatosensory information and accurate visual and vestibular information. Similarly, the platform and eyes state remain the same in condition 6, while the visual surround becomes sway‐referenced, providing not only somatosensory but also visual conflicting information.^30^ Notably, the balance performance and sensory strategy exhibited no such significant correlations in condition 5 that deprived visual input by closing eyes based on condition 4 (Figure 3d). The results indicated that the underlying neural assembly of microstate C modulated the vestibular–visual interaction to handle the balance challenge raised by somatosensory conflict, regardless of the reliability of the visual clues.

However, when it comes to conflicting visual clues in condition 6, the strategy adjustment correlated with the increased occurrence frequency of microstate class C seemed insufficient to significantly improve balance performance. In this condition, the positive correlation of ES6 with the transitions from microstate class A to C (Figure 3c–e) indicated a more significant role of the microstate syntax, which reflecting an encoded sequential activation of neural networks. According to previous studies, microstate class A has been shown to originate from the bilateral superior and middle temporal lobe, associated with specific auditory and visual processing, particularly in spatial visualization tasks.^15^, ^33^, ^34^ The vestibular cortex within temporoparietal association cortex is involved in the visual‐vestibular sensory conflict and plays an important role in the construction of internal model of self‐body, providing fundamental information such as body schema and verticality.^7^, ^39^ The increased transitions from microstate class A to C may imply the increased information interaction between these networks to adjust body scheme and facilitate cognitive processing of anticipatory postural adjustment.

As for the syntax of microstate class A in MD patients, the transitions between class A and B also decreased significantly and showed a negative correlation with the vestibular symptom scales, indicating a positive correlation with vertigo severity (Figure 2e,f, Figure 3a,b). The microstate class B is reported to be associated with visual network and involved in visual–spatial attention and processing.^15^, ^33^, ^40^ Both of the classes are engaged in externally oriented sensory‐cognitive processes, and the binary microstate loop between them has been reported to be dominant in awake and resting‐state^41^ The disruption of direct transition within the binary loop in MD patients may partly due to the hearing loss. But with the lack of correlation with PTA threshold, it could be better explained by the engagement of other cognitive processing during the spontaneously repetitive scanning of sensory input, including the inhibitory control of SN mentioned above. Although the consciously processing could facilitate postural control as we discussed above, it may exacerbate the subjective symptoms at the same time. This would be consistent with the recent study that revealed the role of conscious movement processing in the formation of distorted perceptions of unsteadiness.^42^ The microstate profiles of MD patients may potentially serve as markers for measuring such conscious processing, providing insights into future investigations of potential therapies aimed at optimizing cognitive compensation strategies.

Consistent with our study, the recent rs‐fMRI study in MD patients^10^ reported a similar positive correlation between residual vestibular function and the intranetwork connectivity within the ventral attention network despite different methods for neural network delineation, potentially acting as compensatory mechanisms. Inconsistently, our results did not detect any alterations significantly correlated to PTA threshold or clinical stages determined by hearing loss. The lack of correlation is similar to the microstate findings in patients with idiopathic sudden sensorineural hearing loss,^43^ despite hearing loss being widely believed to cause extensive functional network alterations.^44^, ^45^ This may be attributed to the methodological characteristics of microstate studies, which emphasize the sequential activation of networks and are less sensitive to the functional connectivity between networks.

Different microstate patterns have been raised as potential biomarkers or candidate endophenotype in various neurological and psychological diseases,^16^ including schizophrenia,^19^, ^46^ dementia,^18^, ^47^ and disorders of consciousness.^21^ The affordability and convenience of EEG provide it more chance for clinical applications compared to other neuroimaging modalities. In order to preliminarily explore the potential of microstate profiles as clinical biomarkers for MD patients, we established a SVM classifier, trying to differentiate MD patients from HC and achieving an accuracy of 88.52% with a sensitivity of 86.21%, and a specificity of 90.61% at last. Considering the limited sample size included in this study, the scalability of this result needs further validation in larger datasets in the future. However, it also suggested that microstate features have the potential to serve as biomarkers in the future.

Although numerous microstate analysis reported in literature has provided great support for interpreting the clinical and neuropsychological meaning of microstates, we still should be cautious to interpret the microstate results, especially considering the lack of a full understanding of the functional and pathophysiological details in MD patients. In addition, the affected lateral was not considered in the inclusion criteria, thus the possibility of lateralization issues should be taken into account in further studies. More studies of EEG changes and larger datasets in different MD types might reveal more valuable insights. Longitudinal studies with standard treatment may further elucidate its clinical potentials.

## 5 CONCLUSION

In conclusion, the study first reported the patterns of EEG microstates in patients with MD. The syntax of microstate A and C demonstrated an extra significant conscious movement processing in MD, which may facilitate postural control but aggravate severity of subjective vertigo symptoms. The results provide a further understanding of balance problems in MD and shed a light on potential neurophysiological biomarkers for future MD diagnosis.

## AUTHOR CONTRIBUTIONS

Y.L. and W.L. conceived and designed the study. Y.L., J.L. and W.L. collected the EEG data. P.W., D.Y., Z.C., Z.S., and W.Q. collected the clinical data. Y.L., J.L., and W.L. analyzed the data and wrote the paper together. W.L., W.Q., Y.W., D.Y., Z.C., and H.S. contributed to the manuscript revisions. H.S. administrated the project.

## FUNDING INFORMATION

This work was supported by the National Natural Science Foundation of China (No. 82020108008, No. 82301310, No. 82171147, and No. 82171140).

## CONFLICT OF INTEREST STATEMENT

The authors declare that they have no competing interests.

## Data Availability

The data that support the findings of this study are available from the corresponding author upon reasonable request.
